# Supplementary material for: Rare sex punctuates strict asexual reproduction in the clonal raider ant, Ooceraea biroi
Source: bioRxiv. 2026 Jul 3:2026.07.01.735869. Preprint. [Version 1] doi: 10.64898/2026.07.01.735869 (PMC13345356; doi:10.64898/2026.07.01.735869)
Supplement: Supplement 1 [file NIHPP2026.07.01.735869v1-supplement-1.pdf]

bioRxiv preprint doi: https://doi.org/10.64898/2026.07.01.735869; this version posted July 3, 2026. The copyright holder for this preprint (which was not certified by peer review) is the author/funder, who has granted bioRxiv a license to display the preprint in perpetuity. It is made available under a CC-BY 4.0 International license.

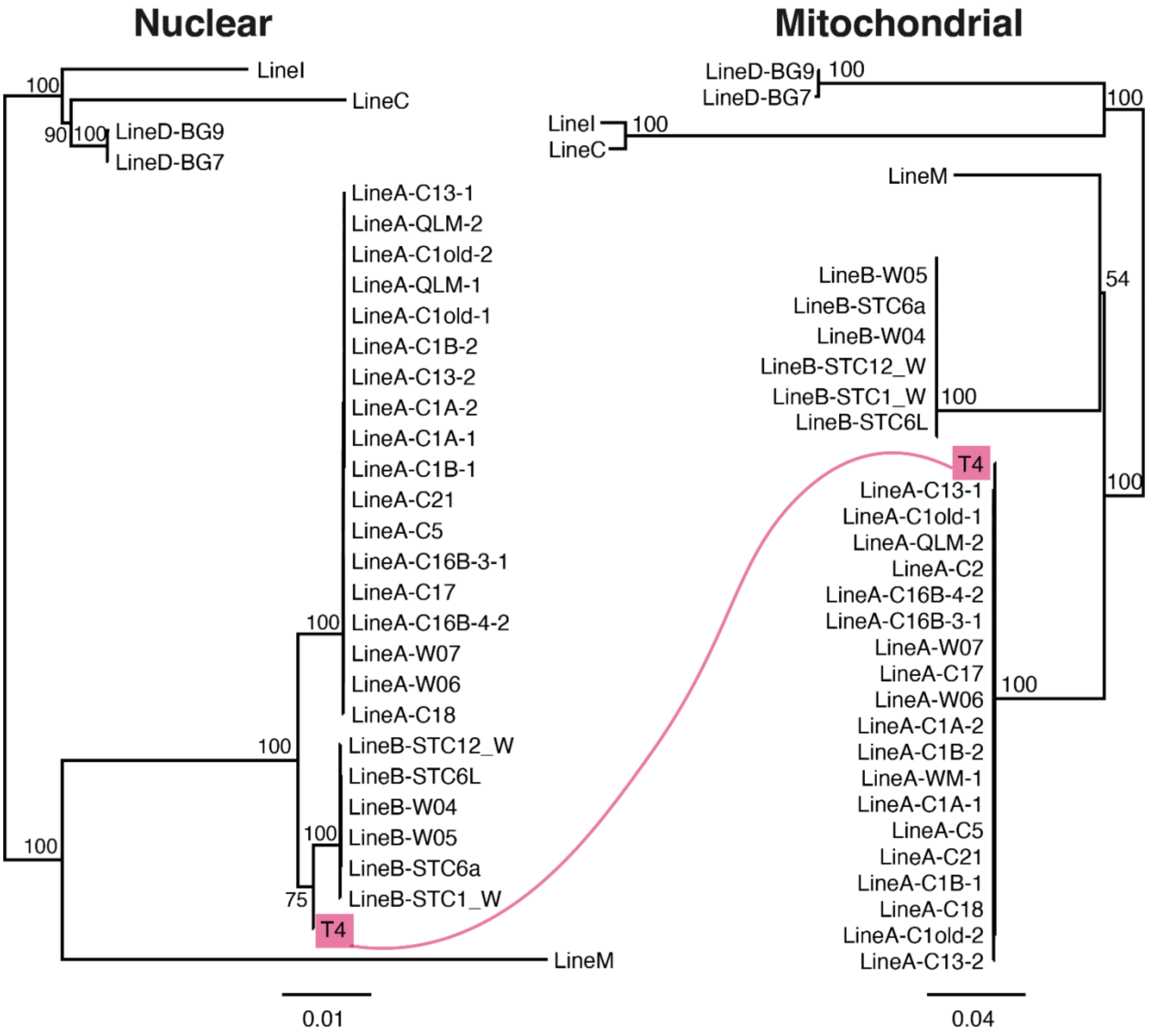

figure S1. Nuclear and mitochondrial maximum likelihood phylogenies for all sequenced *O. biroi* samples. Midpoint-rooted maximum likelihood phylogenies based on SNPs from nuclear and mitochondrial genomes. Bootstrap supports from 1000 bootstrap replicates for major nodes are shown. Scale bars indicate substitutions per site.

bioRxiv preprint doi: https://doi.org/10.64898/2026.07.01.735869; this version posted July 3, 2026. The copyright holder for this preprint (which was not certified by peer review) is the author/funder, who has granted bioRxiv a license to display the preprint in perpetuity. It is made available under a CC-BY 4.0 International license.

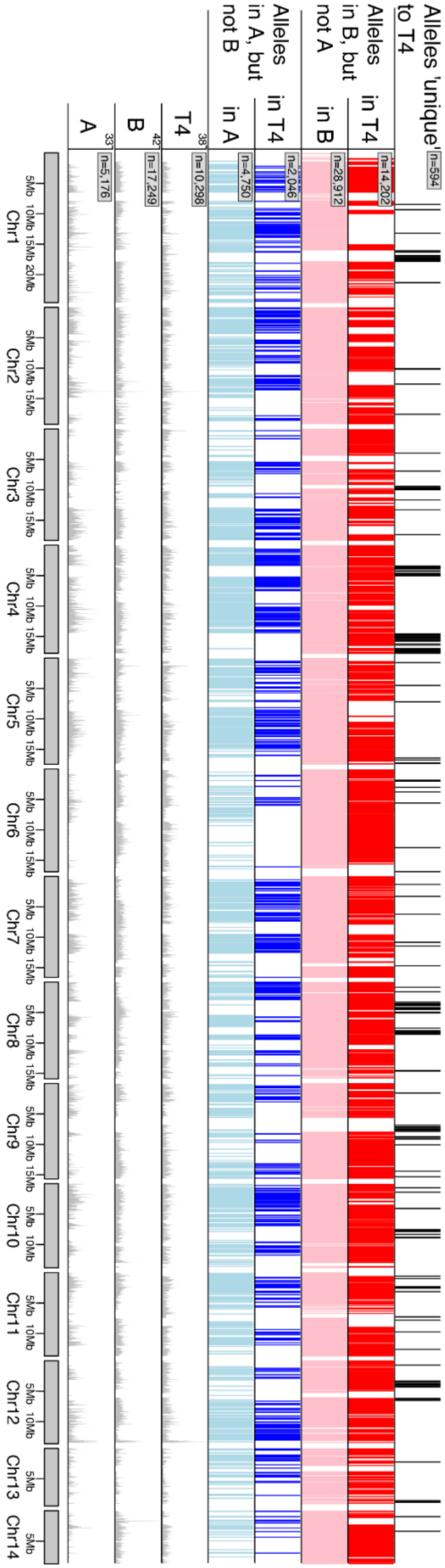

bioRxiv preprint doi: https://doi.org/10.64898/2026.07.01.735869; this version posted July 3, 2026. The copyright holder for this preprint (which was not certified by peer review) is the author/funder, who has granted bioRxiv a license to display the preprint in perpetuity. It is made available under a CC-BY 4.0 International license.

figure S2. Private alleles from clonal lines A and B and their presence in colony T4. Karyoplot depicting, for all chromosomes in the *O. biroi* genome, sites with informative alleles in clonal line A, clonal line B, and colony T4, all shown as vertical tick marks. Heterozygous sites are shown in the gray histograms. For each plot, the number of variants (alleles) is shown in a gray box above chromosome 1.

bioRxiv preprint doi: https://doi.org/10.64898/2026.07.01.735869; this version posted July 3, 2026. The copyright holder for this preprint (which was not certified by peer review) is the author/funder, who has granted bioRxiv a license to display the preprint in perpetuity. It is made available under a CC-BY 4.0 International license.

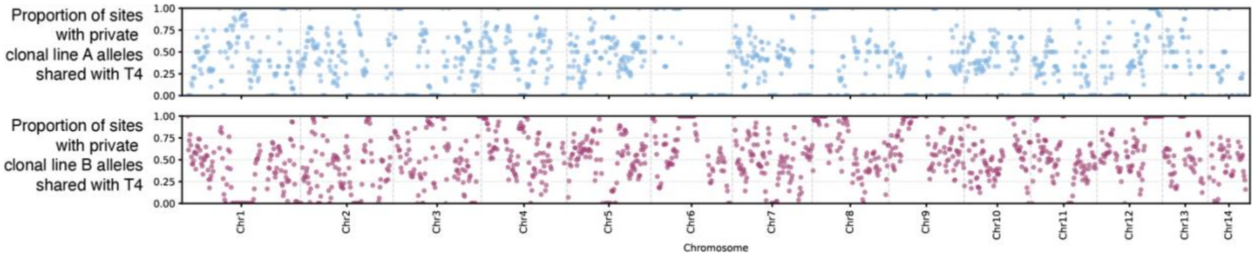

figure S3. Proportions of private alleles from clonal lines A and B that are present in colony T4. Scatterplot depicting, for 600kb windows sliding every 150kb, the proportion of sites with alleles that are otherwise private to clonal line A or B that are shared with colony T4.

bioRxiv preprint doi: https://doi.org/10.64898/2026.07.01.735869; this version posted July 3, 2026. The copyright holder for this preprint (which was not certified by peer review) is the author/funder, who has granted bioRxiv a license to display the preprint in perpetuity. It is made available under a CC-BY 4.0 International license.

433 **table S1. Metadata for DNA sequencing libraries used in this study.**

Sample Name	Clonal Line	Stock Colony	Sex	Ploidy	Published in Study	BioProject
BG9_W	D	BG9	Female	Diploid	Ref. 36	PRJNA1075055
C13-1	A	C13	Female	Diploid	Ref. 35	PRJNA923657
C13-2	A	C13	Female	Diploid	Ref. 35	PRJNA923657
C16B-3-1	A	C16	Female	Diploid	Ref. 30	PRJNA947942
C16B-4-2	A	C16	Female	Diploid	Ref. 30	PRJNA947942
C17	A	C17	Female	Diploid	Ref. 35	PRJNA923657
C18	A	C18	Female	Diploid	Ref. 35	PRJNA923657
C1A-1	A	C1	Female	Diploid	Ref. 35	PRJNA923657
C1A-2	A	C1	Female	Diploid	Ref. 35	PRJNA923657
C1B-1	A	C1	Female	Diploid	Ref. 35	PRJNA923657
C1B-2	A	C1	Female	Diploid	Ref. 35	PRJNA923657
C1old-1	A	C1	Female	Diploid	Ref. 35	PRJNA923657
C1old-2	A	C1	Female	Diploid	Ref. 35	PRJNA923657
C21	A	C21	Female	Diploid	Ref. 35	PRJNA923657
C5	A	C5	Female	Diploid	Ref. 35	PRJNA923657
W06	A	C16	Female	Diploid	Ref. 35	PRJNA923657
W07	A	C16	Female	Diploid	Ref. 35	PRJNA923657
W04	B	STC6	Female	Diploid	Ref. 30	PRJNA947942
W05	B	STC6	Female	Diploid	Ref. 30	PRJNA947942
LineC	C	Unknown	Female	Diploid	Ref. 36	PRJNA1075055
LineD	D	Unknown	Female	Diploid	Ref. 36	PRJNA1075055
LineI	I	Unknown	Female	Diploid	Ref. 36	PRJNA1075055
LineL	M	BG14	Female	Diploid	Ref. 36	PRJNA1075055
MD-B-1-D	B	STC6	Female	Diploid	Ref. 30	PRJNA947942
MD-B-1-M	B	STC6	Female	Diploid	Ref. 30	PRJNA947942
MD-B-7-D1	B	STC6	Female	Diploid	Ref. 30	PRJNA947942
MD-B-7-M	B	STC6	Female	Diploid	Ref. 30	PRJNA947942
STC1_W	B	STC1	Female	Diploid	Ref. 30	PRJNA947942
STC12_W	B	STC12	Female	Diploid	Ref. 30	PRJNA947942
STC6a	B	STC6	Female	Diploid	Ref. 36	PRJNA1075055
STC6L	B	STC6	Female	Diploid	Ref. 30	PRJNA947942
T4	T4	T4	Female	Diploid	This study	PRJNA1277357
WM-1	A	QLM	Female	Diploid	Ref. 35	PRJNA923657
WM-2	A	QLM	Female	Diploid	Ref. 35	PRJNA923657

bioRxiv preprint doi: https://doi.org/10.64898/2026.07.01.735869; this version posted July 3, 2026. The copyright holder for this preprint (which was not certified by peer review) is the author/funder, who has granted bioRxiv a license to display the preprint in perpetuity. It is made available under a CC-BY 4.0 International license.

table S2. Cross-validation (CV) error estimates for ADMIXTURE runs across K values.

CV error values for each run with different K values (numbers of assumed ancestral populations), with lower values suggesting better model fit. The minimum CV error was observed at K = 2, supporting the presence of two major genetic clusters in the dataset.

K	CV Error
1	0.40536
2	0.04607
3	0.07496
4	0.08966
5	0.16603
6	0.16092
